# Canine demodicosis: Hematological and biochemical alterations

**DOI:** 10.14202/vetworld.2020.68-72

**Published:** 2020-01-10

**Authors:** N. Y. Salem, H. Abdel-Saeed, H. S. Farag, R. A. Ghandour

**Affiliations:** 1 Department of Internal Medicine and Infectious Diseases (Internal Medicine), Faculty of Veterinary Medicine, Cairo University, Giza, Egypt; 2 Department of Internal Medicine and Infectious Diseases (Infectious Diseases), Faculty of Veterinary Medicine, Cairo University, Giza, Egypt; 3 Department of Physiology, Faculty of Veterinary Medicine, Cairo University, Giza, Egypt

**Keywords:** antioxidant enzymes, canine generalized demodicosis, C-reactive protein, malondialdehyde, total antioxidant capacity

## Abstract

**Background and Aim:**

One of the most common cutaneous infections seen in veterinary canine practice is canine demodicosis. Demodicosis is a parasitic skin infection with a possible impact on acute-phase proteins (APPs) and oxidant-antioxidant balance. This study aimed to estimate the possible alterations in hematological, biochemical, oxidant-antioxidant, and APP (C-reactive protein [CRP] and albumin) profiles in naturally infected dogs with demodicosis.

**Materials and Methods:**

This study enrolled 21 dogs that were divided into two groups: The control group including 7 apparently healthy dogs and the diseased group including 14 dogs with generalized demodicosis. Demodicosis was confirmed through microscopic detection. Blood samples were collected for the estimation of CBC, total protein, albumin, alanine transaminase, aspartate aminotransferase, blood urea nitrogen, creatinine, superoxide dismutase (SOD), glutathione peroxidase (GPx), total antioxidant capacity (TAC), catalase (CAT), malondialdehyde (MDA), and CRP levels.

**Results:**

Significant reduction in red blood cells along with significant elevation in white blood cells was recorded in the diseased group compared with the control group. There was also significant elevation in MDA, TAC, SOD, and CRP levels along with significant reduction in GSH-Px and CAT levels in the diseased group.

**Conclusion:**

Based on these findings, a relationship between canine generalized demodicosis and oxidant-antioxidant disequilibrium could be suggested. Evidence of this relation manifested in the elevation in MDA and SOD levels and reduction in GPx and CAT levels as a consequence to the release of ROS resulting from *Demodex* infection. CRP elevation is expected in canine demodicosis.

## Introduction

One of the most common cutaneous infections encountered in canine practice is demodicosis [1]. Demodicosis is defined as an inflammatory skin infection of parasitological origin [2]. The disease is caused by *Demodex* mites, mainly *Demodex canis* and to a lesser extent *Demodex injai* [3]. *Demodex* mites are normal flora localized in the skin of most apparently healthy dogs, and the disease arises when these mites overly multiply in the skin and hair follicles [3,4]. Clinically, the disease has two forms, localized and generalized; the latter has more serious outcomes, whereas the former presents with a more favorable prognosis [5]. Clinical picture of the disease is usually associated with erythema, pustules, crusts, hyperkeratosis, and alopecia with secondary pyoderma as a frequent complication [6]. The swiftest means for the diagnosis of canine demodicosis is a microscopic examination of skin scraping as it is both simple and confirmatory [7,8].

Canine demodicosis is an intricate infection, postulated to involve several immunologic and genetic components playing an integral role in its pathogenesis [8]. The level of C-reactive protein (CRP), one of the acute-phase proteins (APPs) associated with non-specific inflammatory reaction in consequence to tissue injury, is believed to increase in canine demodicosis; however, there are limited data available about this [9]. Albumin is classified as a negative APP as it is expected to decrease during the course of an infection [10]. Oxidative stress is a state, in which the production of free radicals surpasses the neutralizing ability of the antioxidant system with consequent tissue damage and possible disruption of molecular structures [5]. Oxidative stress is believed to play a role in numerous human allergic and inflammatory cutaneous infections [11] and canine allergic dermatitis [12].

A link between oxidative stress and demodicosis has been suggested [4]. In recent years, oxidative stress biomarkers were extensively researched for their possible role in the pathogenesis of different diseases. This study aimed to determine the possible alterations in hematological, biochemical, oxidant-antioxidant, and APP (CRP and albumin) profiles in naturally infected dogs with generalized demodicosis.

## Materials and Methods

### Ethical approval

The research procedures were approved by the Institutional Animal Care and Use Committee with document serial number (VetCU0722019062), Faculty of Veterinary Medicine, Cairo University, Egypt.

### Animals and cutaneous examination

This study enrolled 21 dogs of different breeds aged between 9 months and 3 years. The dogs were divided into two groups: The control group including 7 apparently healthy dogs and the diseased group including 14 dogs with dermatologic signs.

The diagnosis was confirmed by microscopic examination of skin scraping [7].

### Sample collection

Blood samples were drawn from the cephalic vein of each animal and distributed into three tubes: Plain, EDTA, and heparin-containing tubes.

EDTA blood was used for hematological estimation and erythrocytes lysate preparation for superoxide dismutase (SOD) and glutathione peroxidase (GPx) tests according to the manufacturer instructions (Biodiagnostic, Egypt).

Plain tube blood was used for serum separation. Serum samples were used to estimate total protein, albumin, alanine transaminase (ALT), aspartate aminotransferase (AST), blood urea nitrogen (BUN), and creatinine using specific test kits (Spectrum Diagnostic, Egypt), malondialdehyde (MDA) (Biodiagnostic, Egypt), and CRP (Vitro Scient, Egypt) [13].

Heparin-containing tubes were used to collect plasma for total antioxidant capacity (TAC) and catalase (CAT) tests (Biodiagnostic, Egypt).

### Statistical analysis

The data were analyzed using Microsoft Excel sheet and results were recorded as mean±standard error. The diseased group data were compared with the control group data using Student’s t-test, SPSS^®^ program ver. 16 (USA). p≤0.05 was considered statistically significant.

## Results

**Figure-1:**
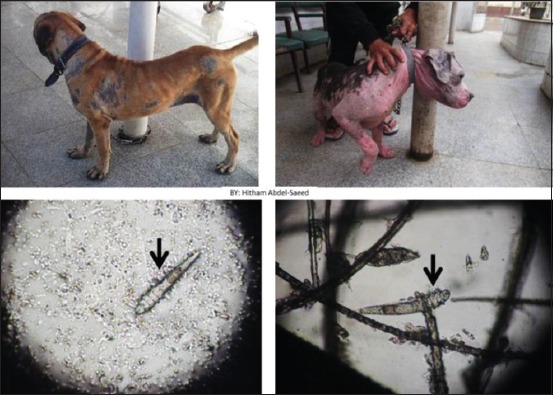
Dogs showing alopecia, erythema, and scaling. Microscopic photo showing cigar-shaped *Demodex*.

Clinically, patients were admitted with signs of erythema, severe itching, alopecia, and scaling. The diagnosis was confirmed by the detection of cigar-shaped organisms under a light microscope (Figure-1).

Hematological alterations are shown in Table-1. A significant reduction in red blood cells (RBCs) associated with a non-significant reduction in packed cell volume (PCV) was recorded. Significant elevation in both white blood cells (WBCs) and eosinophils was recorded in diseased patients compared with control data.

**Table-1:** Hematological alterations in generalized demodicosis patients compared with the control group.

Parameter	Control	Diseased (demodicosis)
PCV (%)	41.8±1.12	39.1±1.88
Hb (g/dl)	13.6±0.38	13.7±0.83
RBCs (×10^6^)	6.58±0.23	5.6±0.25*
MCV (fl)	64.1±1.70	72.7±4.89
MCH (pg)	20.8±0.66	25.4±1.97
MCHC (g/dl)	32.5±0.51	35.2±1.37
WBCs (×10^3^)	12.32±0.58	17.03±1.71*
N%	66.53±3.65	64.3±2.21
L%	23.4±2.11	22.6±1.94
M%	6.92±1.86	6.96±1.58
E%	3.07±0.58	5.68±0.99*
B%	0.0±0.00	0.08±0.08

* p≤0.05. WBCs=White blood cells, MCHC=Mean corpuscular hemoglobin concentration, MCV=Mean corpuscular volume, RBCs=Red blood cells, PCV: Packed cell volume, Hb=Hemoglobin

The biochemical, oxidant-antioxidant, and CRP profiles are presented in Table-2. Statistically significant elevation in total protein and globulin along with a numerical decrease in albumin was recorded in the diseased patients compared with the control dogs. Elevation in ALT and AST was also recorded. A significant increase in MDA, TAC, and SOD levels accompanied by a significant decrease in CAT and GPx were observed in dogs with demodicosis compared with dogs in the control group. CRP activity in diseased dogs was significantly elevated compared with control data.

**Table-2:** Biochemical, oxidant-antioxidant status, and CRP in demodicosis patients compared with the control group.

Parameter	Control	Diseased (demodicosis)
Total protein (g/dl)	6.26±0.12	8.35±0.57**
Albumin (g/dl)	3.2±0.12	2.94±0.24
Globulin (g/dl)	3.05±0.17	5.4±0.69**
A/G ratio	1.12±0.10	0.73±0.16
ALT (IU/L)	11±1.80	19.8±0.07*
AST (IU/L)	15.7±0.53	33.9±4.11***
BUN (mg/dl)	22.2±1.51	19±3.06
Creatinine (mg/dl)	1.05±0.09	0.8±0.11
CAT (U/L)	337.6±19.46	252±16.65**
SOD (U/ml)	175.3±11.4	230.5±24.07*
GPX U/ml)	465.7±26.10	250.6±36.93***
CRP (mg/dl)	7.84±1.10	17±1.98***
MDA (nmol/ml)	2.32±0.14	3.6±0.28***
TAC (mM/L)	0.34±0.02	0.43±0.01**

* p≤0.05,

** p≤0.01,

*** p≤0.001. ALT=Alanine transaminase, SOD=Superoxide dismutase, BUN=Blood urea nitrogen, CAT=Catalase, AST=Aspartate aminotransferase, MDA=Malondialdehyde, CRP=C-reactive protein, TAC=Total antioxidant capacity, GPxGlutathione peroxidase, A/G=Albumin to globulin

## Discussion

In this study, the most consistent clinical signs in patients with demodicosis were erythema, itching, and alopecia. These signs were also recorded in a previous study on canine demodicosis [6]. Diagnosis of the disease mostly depends on skin scraping findings under the microscope [7,8]. Inflammation and irritation caused by mite action in the hair follicles might contribute to the extent of dermatologic lesions and signs [14].

In our study, the hematological profile of affected dogs showed a significant reduction in RBCs associated with insignificant reduction in PCV, whereas leukogram showed elevation in both WBCs and eosinophils. Similar findings were reported by other studies [15,16]. Reduction in feed intake as well as systemic illness consequent to deteriorated health status might contribute to reduction in RBCs [15]. Moreover, loss of skin protein as a result of *Demodex* infection could cause anemia [17]. Extended antigenic stimulation and hypersensitivity reaction subsequent to *Demodex* persistence in tissues could lead to elevated leukocytes and eosinophils [18].

In the present study, significant elevations in total protein and globulin with insignificant decrease in albumin were recorded in generalized demodicosis patients compared with control. Reddy *et al*. [15] reported reduction in albumin and elevation in globulin levels with normal protein level and attributed this to loss of albumin through skin injury. Moreover, Johnson [19] reported that the most common causes of elevated globulin levels in dogs are cutaneous parasitism and pyoderma, with one of these cutaneous parasitic diseases being demodicosis. The non-significant decrease in albumin levels observed is consistent with the findings of Martínez-Subiela *et al*. [9], although the decrease was paradoxically and not statistically significant despite the fact that albumin is considered as a negative APP that changes during inflammation [20]. However, changes in albumin levels do not only result from inflammation *per se* [21]. Significant elevation in ALT and AST levels was observed in dogs with demodicosis in our study, whereas BUN and creatinine levels remained unaltered. These changes could be caused by dermatosis stress [22].

When free radical production surpasses antioxidant capability to neutralize it, oxidative stress ensues [5]. In the present study, an elevated MDA level was recorded in affected dogs compared with the control group. The elevated MDA has been suggested to compensate for antioxidant insufficiency or free radical elevated output. It has also been postulated that the level of lipid peroxidation is associated with disease severity [4]. Infection can trigger the release of pro-oxidative cytokines, leading to the disequilibrium of “transition minerals” (zinc, iron, and copper). These alterations cause Fenton reaction, thereby eliciting damage to the membrane and could be implicated as the cause of MDA elevation [23,24].

In our study, significant increase in SOD was associated with significant decrease in CAT and GPx in affected dogs. SOD modus operandi is to scavenge extra- and intracellular superoxides [25]. It has been suggested that in canine demodicosis, SOD is elevated in response to elevated superoxides release [5]. Moreover, SOD “upregulation” to antagonize free radicals has been proposed [4]. It is a well-known fact that when oxidative damage arises, it is correlated with surplus in endogenous and exogenous antioxidant arm [26]. In the process of elevated SOD activities, H_2_O_2_ production also increases, the body neutralizes the resultant H_2_O_2_ by converting it to H_2_O. Thus, the CAT is the main enzyme for this process, as such reduction in CAT activity is expected [4,5].

In this study, GPx followed CAT pattern in reduction. GPx is known to neutralize ROS [27]. Accentuated nullification of produced free radicals in response to infection could be attributed to the reduction in GPx levels in the patients [4].

There was a significant increase in the TAC in the diseased group compared with the healthy control group. This is in accordance with the findings of Martínez-Subiela *et al*. [9], who reported an increased TAC in canine demodicosis patients. The cause of this elevation was attributed to the elevation in SOD activity that represents “first-line defense antioxidant” [28]. In the present study, GPx and CAT activities were reduced, and both are endogenous antioxidants [29]. Moreover, in other animal disease models, Hassanpour *et al*. [30] reported a positive correlation between CAT and TAC, and both decrease simultaneously in *Theileria*-infected cattle.

Positive APPs represented in this study in the form of CRP were significantly increased. Demodicosis is postulated to elicit inflammatory reaction with the release of cytokines into the bloodstream that consequently leads to a rise in CRP levels [21].

## Conclusion

Based on these results, a relationship between canine generalized demodicosis and oxidant-antioxidant disequilibrium is suggested. Evidence of this relation manifested in the elevation in MDA, TAC and SOD levels and reduction in GPx and CAT levels as a consequence to ROS released due to *Demodex* infection. Elevation in CRP level is expected in canine demodicosis.

## Authors’ Contributions

NYS, HA, HSF, and RAG: Formulated and designed the idea. HSF: Physical and parasitological analysis. NYS and HA: Samples collection and processing. RAG: Data collection. NYS and HA: Writing the first draft. HSF and RAG: Revision. All authors read and approved the final manuscript.
